# Trends in research productivity among obstetrics and gynecology residency applicants in the United States: a national comparison across specialties, 2016 to 2024

**DOI:** 10.1080/10872981.2026.2739701

**Published:** 2026-11-04

**Authors:** Shea E. Randall, Emily Martinbianco, Katherine Sadak, Anne M. Tjaden Peiffer

**Affiliations:** a Loyola University Chicago Stritch School of Medicine, Maywood, IL, USA; b Cleveland Clinic Department of Obstetrics and Gynecology, Cleveland, OH, USA; c Loyola University Medical Center Department of Obstetrics and Gynecology, Maywood, IL, USA

**Keywords:** Residency match, research productivity, residency selection, obstetrics and Gynecology, national resident matching programme

## Abstract

**Background:**

Research productivity has become an increasingly emphasised component of residency selection. However, longitudinal trends in obstetrics and gynecology (OB/GYN) applicant research output relative to other specialties remains poorly characterised, particularly in the context of evolving evaluation practices and the transition of USMLE Step 1 to pass/fail scoring.

**Objective:**

To evaluate trends in research productivity among matched OB/GYN applicants and compare these patterns with other medical and surgical specialties from 2016 to 2024.

**Methods:**

A retrospective analysis of the National Resident Matching Program (NRMP) *Charting Outcomes* data from 2016–2024 was performed. Mean research experiences and research items (abstracts, posters, and publications) reported by matched applicants were extracted for each specialty and year. Descriptive longitudinal trend analyses were performed to evaluate changes over time. Mean research experiences and research items (abstracts, posters, and publications) were summarized by specialty and year, and linear trend lines were used to illustrate directional patterns. Comparisons with other specialties (all specialties, surgical specialties, and specialties with similar match rate ± 5%) were conducted descriptively.

**Results:**

From 2016 to 2024, matched OB/GYN applicants saw increased mean research items (4.2 to 9.0; +114%) and research experiences (3.2 to 3.8; +19%). Growth in research output demonstrated upward trends across OB/GYN and other specialties, with increases observed across both surgical and non-surgical fields. Although unmatched applicants showed similar relative increases, matched applicants consistently reported higher absolute research output.

**Conclusions/implications:**

OB/GYN applicants have increased their research output in recent years like other surgical and similarly competitive fields. As programs increasingly rely on scholarly metrics, educators and residency leaders should consider strategies to ensure equitable access to research opportunities and maintain holistic review practices during resident selection.

## Introduction

Residency selection in the United States has become increasingly competitive, with growing emphasis on research productivity as a differentiating factor among applicants. For graduate medical education (GME) leaders and educators, understanding how applicant evaluation metrics evolve over time is essential to ensuring fair, holistic, and mission-aligned selection processes. In Obstetrics and Gynecology (OB/GYN), a specialty marked by rapid growth in applicant volume, shifts in evaluation criteria may have meaningful implications for both trainees and training programs

The National Resident Matching Program (NRMP) data demonstrate a steady rise in the number of OB/GYN residency applicants over the past decade, accompanied by increases in academic metrics such as United States Medical Licensing Examination (USMLE) scores and reported research items and experiences [1,2]. Between 2016 and 2024, OB/GYN applicants increased from 1606 to 2034, while the mean number of research items reported by matched applicants more than doubled compared with earlier cohorts [2,3]. Prior studies and surveys of program directors suggest that scholarly output has become an increasingly influential factor in residency selection, reflecting broader trends across multiple medical specialties [4,5].

This shift may have been further amplified by the 2022 transition of the USMLE Step 1 examination from numeric scoring to pass/fail. Although this change was intended to promote holistic review, the reduced emphasis on standardized test scores may have inadvertently increased reliance on other quantifiable metrics, including research productivity [6]. While rising research output has been documented within OB/GYN and other individual specialties, no prior study has directly compared longitudinal trends in OB/GYN applicant research productivity with those of other specialties using multi-year NRMP data, particularly including the post-Step 1 pass/fail era.

The objective of this study is to evaluate changes in research productivity among matched OB/GYN residency applicants from 2016 to 2024 and to compare these trends with those observed in other medical specialties.

## Methods

A retrospective analysis was performed using publicly available data from the NRMP *Charting Outcomes in the Match* reports from 2016 to 2024 [1]. These annual reports summarize applicant characteristics by specialty, including the mean number of research experiences and mean number of abstracts, posters, and publications (research items), Step 1 and Step 2 scores, and Alpha Omega Alpha (AOA) status, among other criteria. These reports reflect only applicants verified by NRMP and are widely used in research on residency competitiveness and outcomes. Institutional review board approval was obtained from Loyola University Chicago.

All matched and unmatched U.S. Allopathic senior applicants were included, as reported in the NRMP tables. For each specialty and year, the following variables were extracted: match rate, mean number of research experiences, mean number of research items (abstracts, posters, and publications), mean Step 1 and Step 2 scores, percentage of AOA members, and percentage of graduates from the 40 US medical schools with the highest NIH funding. Data were compiled in spreadsheet format (Google Sheets) and analysed using R version 4.3.1 (R Foundation for Statistical Computing, Vienna, Austria).

Given the limited number of time points available in NRMP Charting Outcomes reports (2016–2024), analyses were intentionally limited to descriptive longitudinal trend assessments. For each specialty and year, mean research experiences, mean research items (abstracts, posters, and publications), and match rates were recorded. Percent change over time was calculated to characterize the magnitude of change across specialties.

Linear trend lines were generated to visualize directional changes in research productivity over time. However, no inferential statistical testing of slope differences was performed due to the small number of observations per specialty and the use of aggregated specialty-level data. Because the analysis was purely descriptive, no parametric or nonparametric inferential statistical tests were performed, and no formal model specifications were defined.

Comparator groups were defined a priori as fixed categorical groupings based on specialty characteristics: (A) all specialties, (B) surgical specialties (general surgery, neurosurgery, orthopedic surgery, otolaryngology, plastic surgery, and vascular surgery) including OB/GYN for comparison, and (C) specialties with match rates within ±5% of OB/GYN in the corresponding year. These groupings were used solely for descriptive comparison and were not derived from statistical clustering or outcome-based modelling. Surgical specialties were analyzed as a distinct comparator group given known differences in competitiveness and historically higher emphasis on scholarly productivity in residency selection within surgical fields compared with non-surgical fields. The ±5% threshold was selected a priori as a pragmatic range to identify specialties with broadly similar historical match competitiveness to OB/GYN, allowing for contextual comparison without imposing overly restrictive grouping data. No slope estimation or formal statistical comparison of trends between comparator groups was performed; comparisons across groups were limited to descriptive visualization of longitudinal patterns. Analyses were not weighted by specialty size/applicant volume, as only aggregated mean values were available at the specialty level in NRMP reports. Because the dataset consists of repeated cross-sectional specialty-level summaries rather than individual-level longitudinal observations, repeated measures modelling was not applicable.

## Results

A total of twenty-two specialties were included in the study. All comparisons across specialties are presented as descriptive patterns based on aggregated NRMP data and should not be interpreted as inferential differences. In 2016, the mean number of research experiences among all matched applicants ranged from 2 (family medicine) to 8.4 (physical medicine and rehabilitation), while the mean number of abstracts, posters, and publications ranged from 2.6 (family medicine) to 13.4 (neurosurgery). By 2024, the mean number of research experiences among all matched applicants ranged from 2.1 (family medicine) to 8.6 (plastic surgery), while the mean number of abstracts, posters, and publications ranged from 4.2 (family medicine) to 37.4 (neurosurgery). Over the same period, match rates in 2016 ranged from 71% (vascular surgery) to 98% (internal medicine and diagnostic radiology), while in 2024 they ranged from 68.7% (neurosurgery) to 99.7% (paediatrics). The match rate for OB/GYN applicants ranged from 83.9% (2022) to 91% (2016) and was 85.7% in 2024.

In 2024, specialties with the highest research experiences and mean abstracts, posters, and publications included plastic surgery (8.6; 34.7), orthopedic surgery (8.1; 23.8), otolaryngology (7.1; 20), dermatology (6.4; 27.7), and neurosurgery (5.8; 37.4) (Table 1). In contrast, specialties with the lowest values were family medicine (2.1; 4.2), pediatrics (2.6; 6.4) emergency medicine (2.8; 5.7), psychiatry (3; 7.5), and child neurology (3; 9.8) (Table 1). These descriptive differences highlight a wide range of research engagement across specialties.

**Table 1.** Match rate, research items, and research experiences among all specialties, 2024.

Specialty	Match rate	Matched abstracts posters publications	Unmatched abstracts posters publications	Matched mean research experiences	Unmatched mean research experiences
Anesthesia	85.2	9	5.4	3.8	3.1
Child Neurology	97.1	9.8	1	3	2
Dermatology	70.5	27.7	19	6.4	4.9
Diagnostic Radiology	86.4	12	8	4.4	3.6
Emergency Medicine	98	5.7	5	2.8	2.3
Family Medicine	98.8	4.2	1.4	2.1	1.8
General Surgery	81.8	10.9	7.3	4.2	3.7
Internal Medicine	97.8	8.7	6.2	3.3	3.3
Internal Medicine/Pediatrics	86	6.9	6.2	3.1	2.6
Interventional Radiology	82.8	15.8	10.1	4.7	4.6
Neurosurgery	68.7	37.4	31.8	5.8	5.5
Neurology	93.8	8.8	5.5	3.5	2.8
OB/GYN	85.7	9	6.8	3.8	3.3
Orthopedics	73.1	23.8	18	8.1	8
Otolaryngology	81.9	20	15.6	7.1	5.5
Pathology	93.5	8.4	4.1	3.1	2.3
Pediatrics	99.7	6.4	21.5	2.6	0.5
Physical Medicine & Rehabilitation	84.8	8.6	6.4	3.4	3.3
Plastics	74.3	34.7	26.3	8.6	9.2
Psychiatry	89.1	7.5	4.6	3	3.1
Radiation Oncology	97.5	15.9	13.5	4.2	2
Vascular Surgery	88.5	12.8	8	4.6	4.5

From 2016 to 2024 in OB/GYN specifically, the mean number of abstracts, posters, and publications among matched applicants increased from 4.2 to 9, while mean research experiences increased from 3.2 to 3.8. Over the same period, the match rate decreased from 91% to 85.7%, suggesting increasing research output alongside a modest decline in match success.

### Mean research experiences over time

From 2016 to 2024, OB/GYN applicants demonstrated a gradual increase in the mean number of research experiences (Figure 1). When compared descriptively with other specialties, upward trends were observed across the broader residency applicant pool. Surgical specialties including general surgery, neurosurgery, orthopedic surgery, otolaryngology, plastic surgery, and vascular surgery demonstrated obvious increasing research experiences over time. When including OB/GYN in this group for the sake of comparison, there was a combined mean annual increase of *β* = 0.25. Within this group, OB/GYN demonstrated a more modest trajectory (*β* = 0.11) compared to other surgical specialties such as orthopaedic surgery (*β* = 0.50) and plastic surgery (*β* = 0.44). Despite these differences in magnitude, all specialties showed increases over time, and OB/GYN followed a similar overall directional trend.

**Figure 1.**
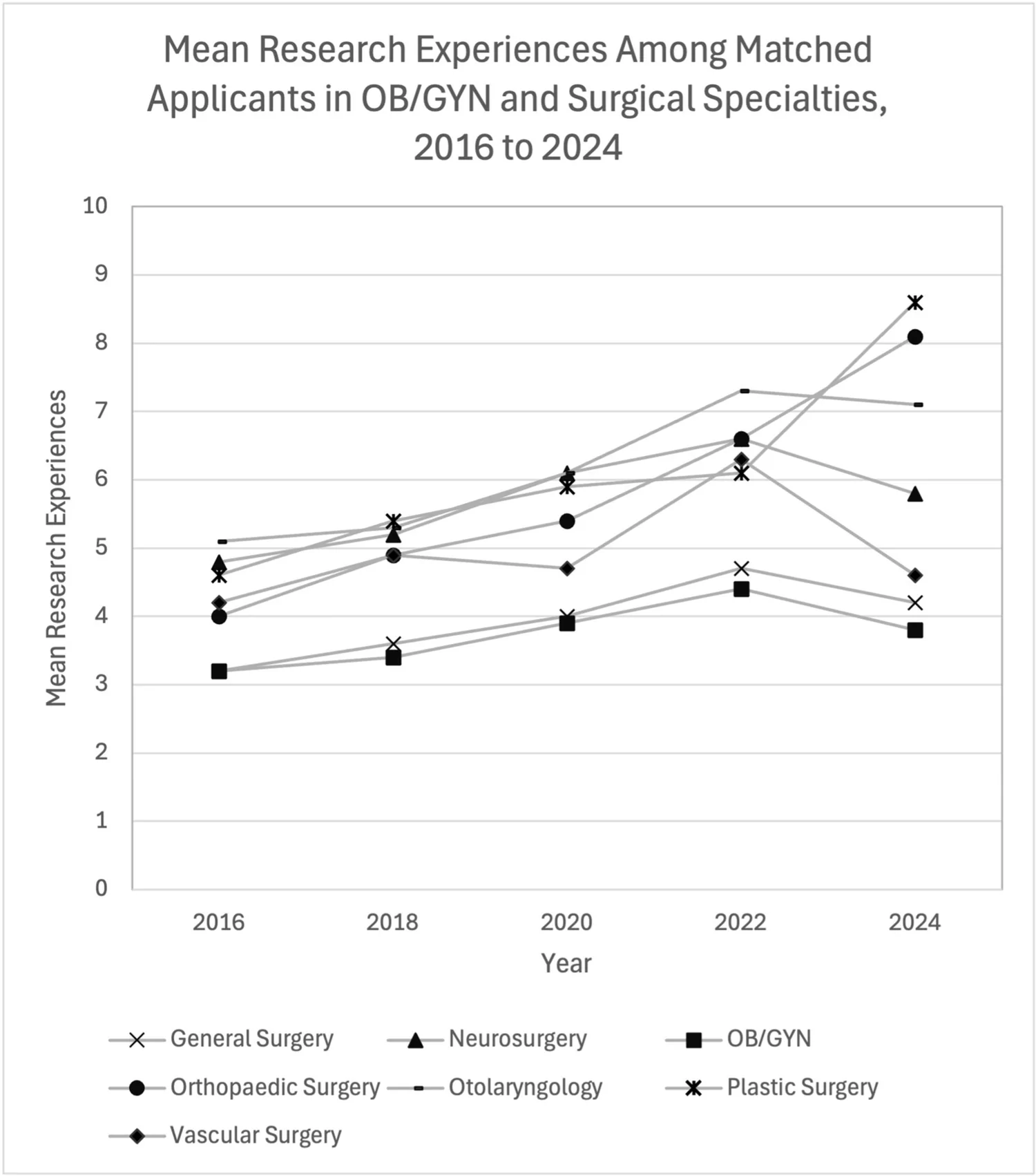
Mean research experiences among matched applicants in OB/GYN and surgical specialties, 2016 to 2024. Mean number of research experiences is shown for OB/GYN applicants and comparator surgical from 2016 to 2024. Data points represent yearly means. Linear regression was used to evaluate trends over time.

### Mean abstracts, posters, and publications over time

From 2016 to 2024, OB/GYN applicants demonstrated a marked increase in the mean number of abstracts, posters, and publications (Figure 2). Similar upward trends in research output were observed across other specialties, including surgical fields. Across surgical specialties, the mean annual increase in research output was *β* = 1.58, reflecting a strong and consistently increasing trajectory over time. This trend was driven in part by more accelerated growth in fields such as neurosurgery (*β* = 2.76) and plastic surgery (*β* = 2.99), whereas OB/GYN demonstrated a more moderate increase (*β* = 0.575). When compared descriptively with specialties of similar competitiveness (defined as a match rate ±5% of OB/GYN), patterns of growth appeared comparable, suggesting that increases in research output are not unique to OB/GYN but reflect broader trends across the residency applicant pool.

**Figure 2.**
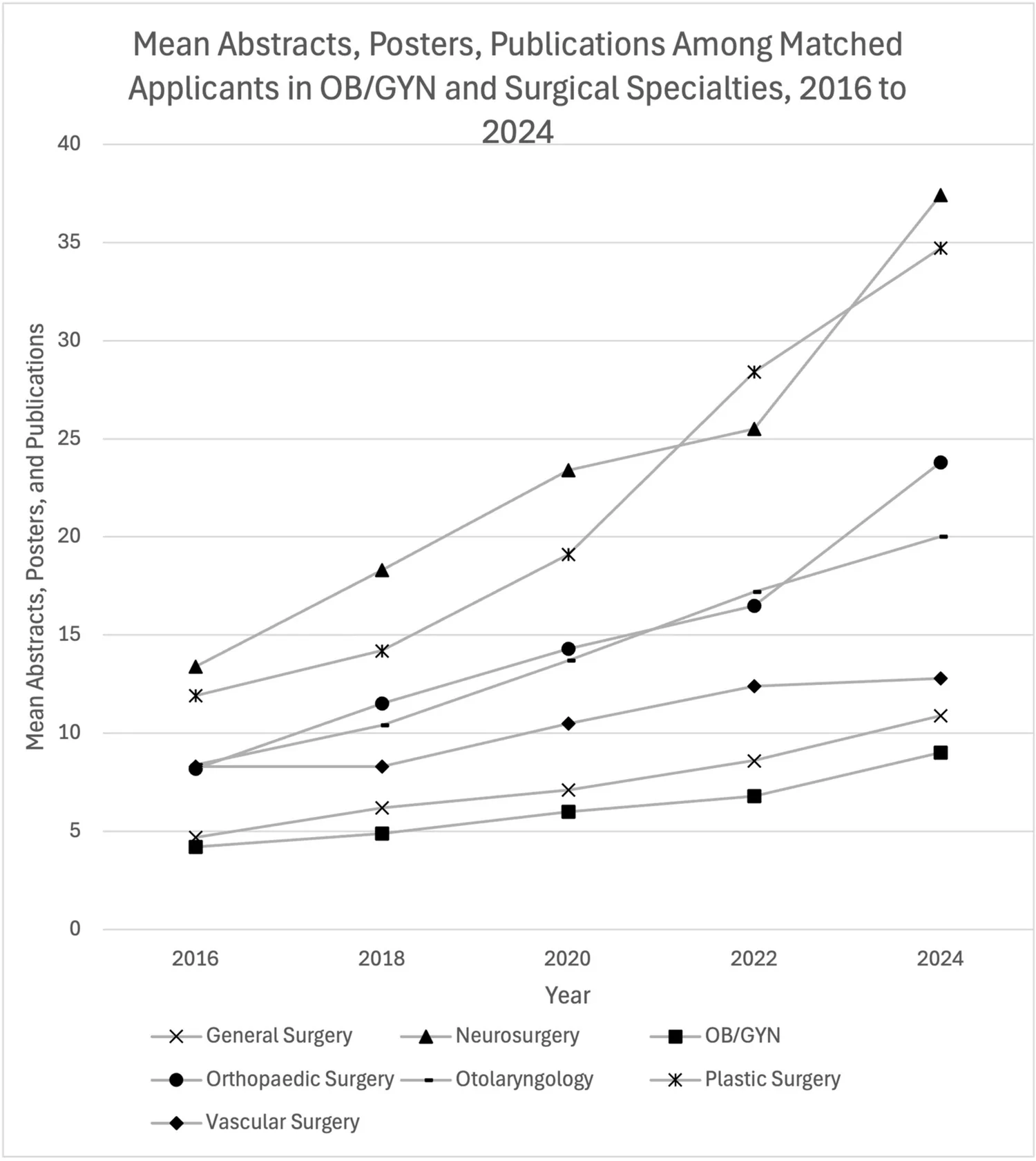
Mean abstracts, posters, publications among matched applicants in OB/GYN and surgical specialties, 2016 to 2024. Mean number of abstracts, posters, and publications is shown for OB/GYN applicants and comparator surgical specialties from 2016 to 2024. Data points represent yearly means. Linear regression was used to assess trends over time.

### Research output compared with specialties of similar competitiveness

When comparing OB/GYN applicants with a predefined group of specialties with similar match competitiveness (match rate ±5% of OB/GYN), including anesthesia, diagnostic radiology, general surgery, internal medicine/pediatrics, interventional radiology, otolaryngology, physical medicine and rehabilitation, psychiatry, and vascular surgery, similar upward trends in research output were observed across specialties. Although the magnitude of increase varied, OB/GYN demonstrated an upward trend in research output over time that was observed across all comparator specialties.

### Research output relative to match competitiveness across surgical specialties and OB/GYN

Across surgical specialties, the ratio of research output (mean abstracts, posters, and publications) to match rate demonstrated a consistent upward trend over time (mean *β* = 0.023), indicating increasing academic productivity relative to competitiveness. This effect was most pronounced in highly competitive fields such as plastic surgery (*β* = 0.046) and neurosurgery (*β* = 0.043), while obstetrics and gynecology (*β* = 0.007) and vascular surgery (*β* = 0.006) exhibited more modest growth. Collectively, these findings reflect a steady rise in research expectations relative to match selectivity across surgical disciplines and OB/GYN.

### Research productivity in matched vs. unmatched OB/GYN applicants

To further explore factors associated with successful matching, research output was compared between matched and unmatched U.S. allopathic OB/GYN applicants from 2016 to 2024. Matched applicants experienced a 114% increase in research items (abstracts, posters, and publications), from a mean of 4.2 in 2016 to 9.0 in 2024. Unmatched applicants experienced a slightly higher percent increase of 135%, from 2.9 to 6.8 research items over the same period (Figure 3). In contrast, growth in research experiences was similar between groups, with matched applicants increasing 19% and unmatched applicants increasing 18% from 2016 to 2024 (Figure 4). These findings suggest that although both matched and unmatched applicants have increased research output over time, matched applicants maintain a higher absolute number of research items.

**Figure 3.**
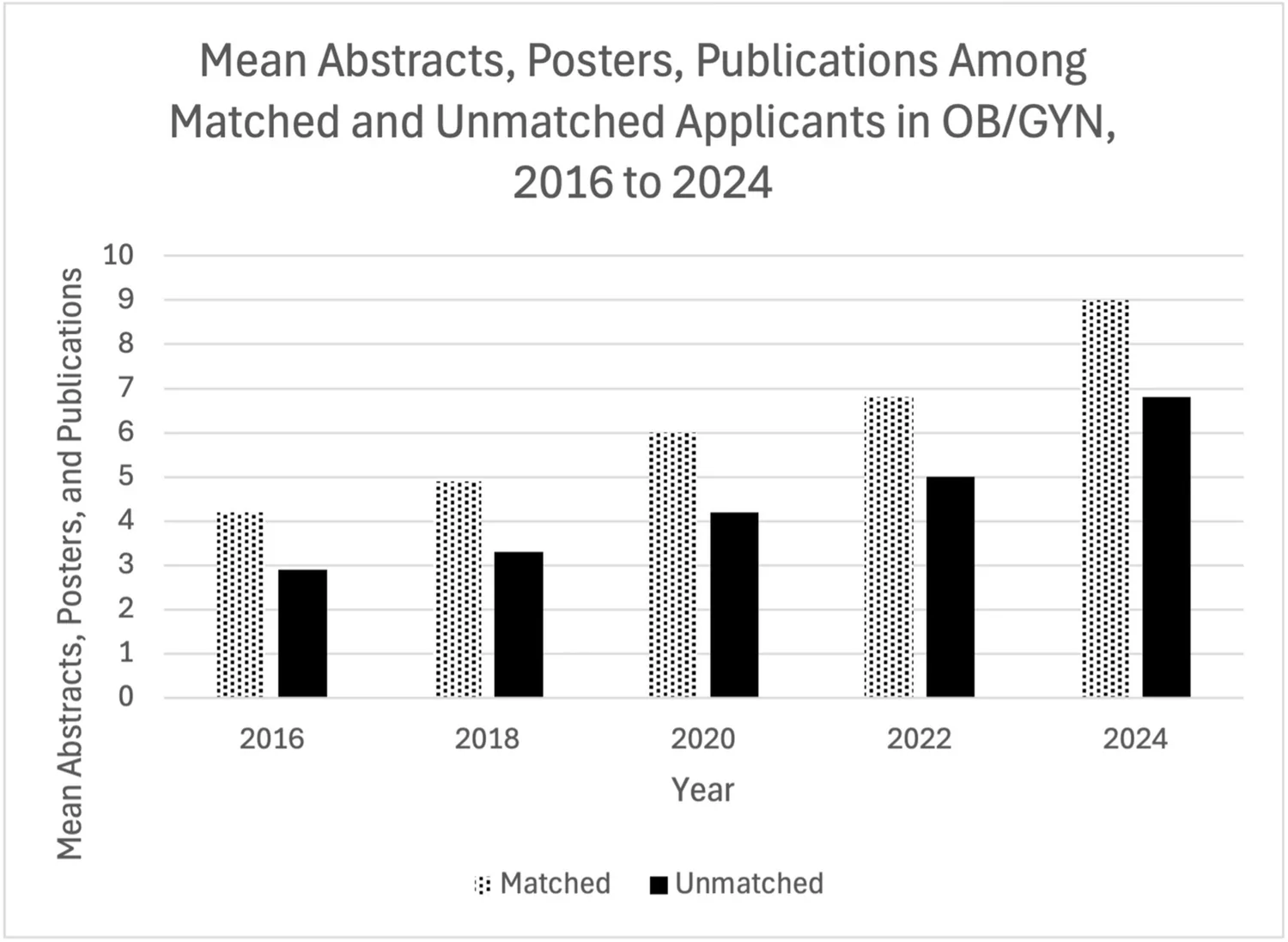
Mean abstracts, posters, publications among matched and unmatched applicants in OB/GYN, 2016 to 2024. Mean number of abstracts, posters, and publications is shown for matched and unmatched OB/GYN applicants from 2016 to 2024. Data points represent yearly means.

**Figure 4.**
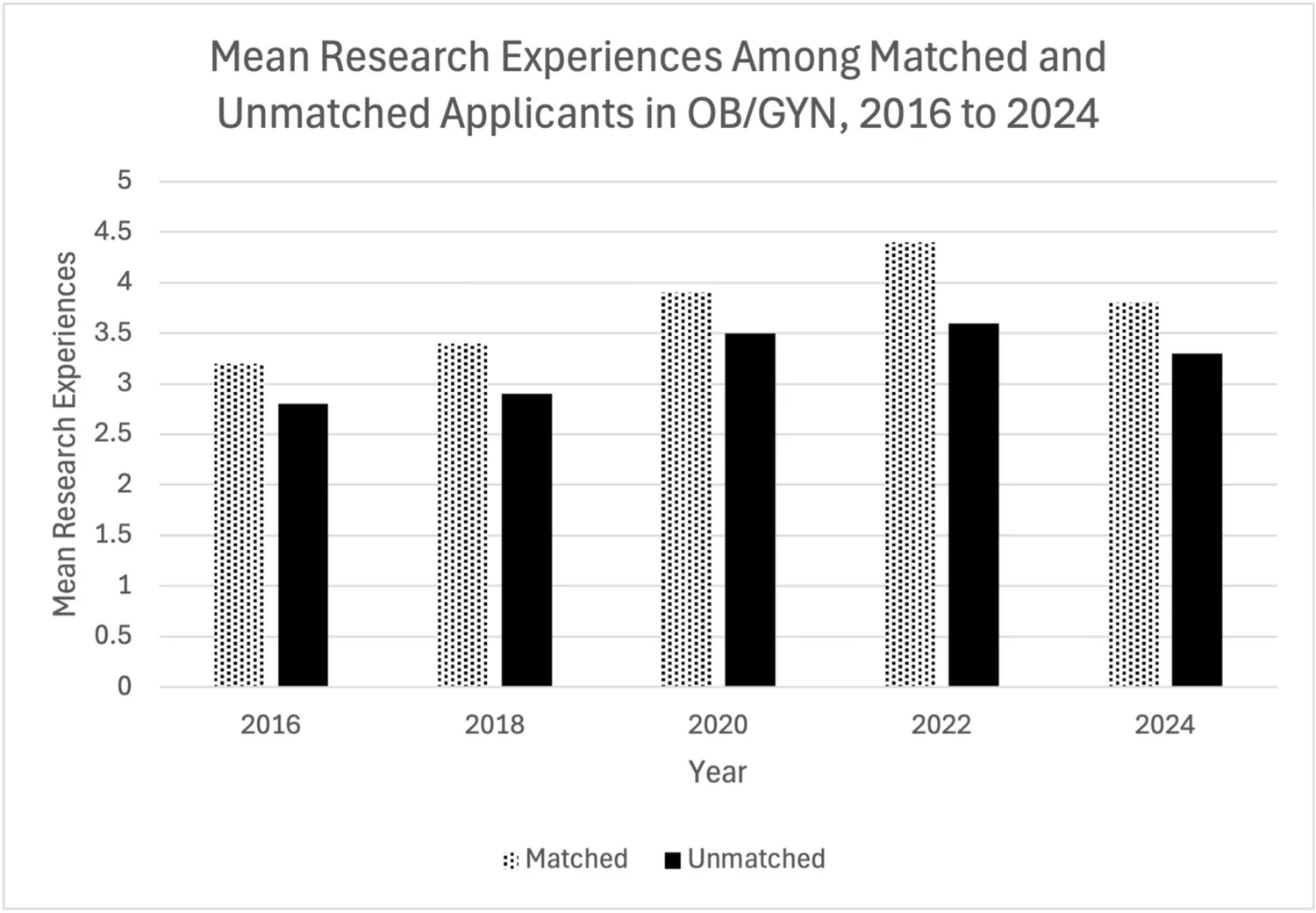
Mean research experiences among matched and unmatched applicants in OB/GYN, 2016 to 2024. Mean number of research experiences is shown for matched and unmatched OB/GYN applicants from 2016 to 2024. Data points represent yearly means.

## Discussion

Given the aggregated and descriptive nature of the NRMP dataset, comparisons across specialties should be interpreted as descriptive patterns rather than inferential differences. This study demonstrates a marked increase in reported research output among matched OB/GYN residency applicants between 2016 and 2024, broadly consistent with descriptive trends observed across other surgical and similarly competitive specialties. The magnitude and consistency of this increase suggest that scholarly productivity has become an increasingly prominent feature of residency competitiveness in the current match environment. However, the steady escalation of research expectations raises important questions about equity, access to research opportunities, and merit in the residency application process.

These findings align with prior reports indicating rising research expectations across graduate medical education, particularly in specialties with growing applicant volume and limited training positions. Similar increases in applicant research output have been described in other specialties such as orthopedics and otolaryngology, suggesting that this trend is not unique to OB/GYN but rather reflective of broader structural shifts in residency selection [7,8]. The transition of the USMLE Step 1 examination to pass/fail scoring in 2022 may have further contributed to shifts in residency selection emphasis across specialties. Descriptively, increases in research output were observed both before and after the transition. However, a formal pre-/post-Step 1 pass/fail comparison or segmented regression analysis were not performed due to the limited number of aggregated time points and the inability to reliably identify structural breakpoints within specialty-level summary data. It is likely that multiple concurrent factors, including increasing applicant volume, evolving program director expectations, and expansion of research years and virtual collaborations have collectively influenced these trends. While the Step 1 transition represents an important structural change in medical education, its specific contribution to observed increases in research productivity cannot be isolated within the current dataset. That said, as standardized numeric metrics diminish in prominence, program directors across specialties have reported greater reliance on alternative indicators, including factors such as research involvement and scholarly output [9]. While intended to support holistic review and promote student wellness, this shift may unintentionally emphasise metrics that are more readily attainable in certain institutional contexts [10].

Importantly, increased research productivity does not occur in a vacuum. Access to mentorship, protected time, funding, and active research infrastructure varies substantially across medical schools. Applicants from research-intensive institutions or departments with established OB/GYN research programs may have greater opportunities to participate in publishable work. Students from smaller programs, osteopathic or international schools, or institutions with limited specialty-specific research may face structural barriers [9]. As a result, rising research expectations may amplify existing inequities in the residency application process, particularly for students who may be balancing caregiving responsibilities, employment, or other non-academic obligations. In a specialty that emphasizes equity, reproductive justice, and patient-centred care, these dynamics warrant deliberate action by educators and program leaders.

Several factors may influence the interpretation of these findings. Because NRMP *Charting Outcomes in the Match* data are self-reported and aggregated at the specialty level, findings are subject to ecological limitations and cannot capture individual-level variation in research productivity, institutional resources, or scholarly impact. The absence of demographic and institutional variables in this analysis further limits the ability to evaluate how research access differs by applicant background or training environment. Additionally, specialty-level averages obscure within-specialty variability and may mask trends within subgroups. Although the temporal association with the USMLE Step 1 transition to pass/fail is notable, other concurrent changes in medical education such as popularity of research years, virtual collaborations, or heightened applicant anxiety may also contribute to these observed patterns.

Future research should examine individual-level applicant data to better characterize how research productivity intersects with institutional resources, applicant demographics, and match outcomes. Qualitative studies exploring applicant and program director perspectives on the role of research in residency selection may further clarify how scholarly activity is interpreted and valued. Longitudinal analyses incorporating additional post-Step 1 pass/fail match cycles will be essential to determine whether these trends persist and to inform evidence-based approaches to equitable residency selection.

## Educational implications

These findings have practical implications for residency selection and undergraduate medical education. As research metrics become increasingly prominent, program directors, selection committees, and student advisors may inadvertently privilege applicants from resource-rich institutions rather than those with comparable potential. Programs should consider incorporating structured holistic review processes, contextualizing research output to relative opportunity, and explicitly valuing diverse forms of scholarship and service. At the medical school level, expanding equitable access to mentorship, protected research time, and research infrastructure is necessary to mitigate disparities in competitiveness. Aligning expectations with accessible opportunities will be critical to ensuring that rising research benchmarks do not unintentionally narrow diversity within the OB/GYN workforce.

## Conclusion

Research productivity among matched OB/GYN applicants has increased substantially from 2016 to 2024, which appears consistent with descriptive patterns observed across other surgical and similarly competitive specialties. Although this reflects a broader shift toward scholarly achievement, increasing reliance on research output as a marker of competitiveness risks exacerbating inequities in access and opportunity. Residency programs and medical schools must adopt intentional, holistic evaluation and expand equitable mentorship and research access to preserve diversity and fairness within the OB/GYN workforce.

## Data Availability

Data used in this study are publicly available through the National Resident Matching Program Charting Outcomes in the Match reports. Extracted datasets generated during the current study are available from the corresponding author upon reasonable request.

## Presentation

*This work was presented as a poster at the CREOG and APGO 2026 Annual Meeting in Seattle, Washington from February 18-21, 2026.
